# Probing the Relationship Between Perioperative Complications in Patients With Valvular Heart Disease: Network Analysis Based on Bayesian Network

**DOI:** 10.2196/68710

**Published:** 2025-10-07

**Authors:** Wenyuan Lu, Kun Zhu, Zhiliang Gao, Yuanming Li, Hanwei Tang, Cheng Sun, Jianfeng Hou

**Affiliations:** 1 Fuwai Hospital, Chinese Academy of Medical Sciences, Peking Union Medical College, No. 167, North Lishi Street, Beijing, 100037, China, 86 15321663719

**Keywords:** perioperative complications, valvular heart disease, bayesian network, network analysis, probability inference

## Abstract

**Background:**

Heart valve surgery is associated with a high risk of perioperative complications. However, current approaches for predicting perioperative complications are all based on preoperative or intraoperative factors, without taking into account the fact that perioperative complications are multifactorial, dynamic, heterogeneous, and interdependent.

**Objective:**

We aimed to construct and quantify the association network among multiple perioperative complications to elucidate the possible evolution trajectories.

**Methods:**

This study used the data from China Cardiac Surgery Registry (CCSR), in which 37,285 patients were included in the analysis. A Bayesian network was used to analyze the associations among 12 complications. Score-based hill-climbing algorithms were used to build the structure and the association between them was quantified using conditional probabilities.

**Results:**

We obtained the network of valve surgery complications. A total of 13 nodes represented complications or death, and 34 arcs with arrows represented the directly dependent relationship between them. We identified clusters of complications that were logically related and not related and quantified the associations. The correlation coefficient between complications increases with the severity of the complications, ranging from 0.01 to 0.41. Meanwhile, the probability of death when multiple complications occurred was calculated. Even mild complications, when progressing to multiple organ dysfunction syndrome, result in a mortality rate of over 90%.

**Conclusions:**

Our network facilitates the identification of associations among specific complications, which help to develop targeted measures to halt the cascade of complications in patients undergoing the valve surgery.

## Introduction

Valvular heart disease (VHD) is a leading cause of cardiovascular morbidity and mortality and affects >2% of the population [1]. Its treatment mainly relies on valve repair and replacement. The incidence of perioperative complications is estimated to remain as high as 10.7% [2]. The complications translate into prolonged hospital stays, additional health care costs, and increased mortality. Therefore, many studies have evaluated predictors of perioperative complications. Current approaches for identifying the significant risk factors associated with perioperative complications are mainly through traditional machine learning models. The risk factors involved in these models are all preoperative factors, and the perioperative complications are regarded as independent events, which ignore the consequences of complications. There is growing evidence that perioperative complications are not independent, and the first complications could lead to secondary complications or even death [3,4], such as retained blood within the pericardium increasing the risk of postoperative atrial fibrillation (AF) [5,6] and acute renal failure related to cardiac arrest and death, similar to the domino effect [3]. The evidence suggested that most perioperative complications are multifactorial, dynamic, and context-specific. It is likely that perioperative complications form a complex system contributing to progressive physiological and biochemical deterioration. However, traditional approaches missed some differences in the trajectory of occurrence from initial complications to death [7] and failed to chart a comprehensive landscape of perioperative complications. A better understanding of the development pattern of perioperative complications is the focus of surgical quality improvement initiatives and an ongoing challenge.

The complex relationships among perioperative complications could provide strategies to improve prediction of further outcomes and inform the target and timing of interventions. Meanwhile, the difficulties in estimating the likelihood that a certain complication is secondary to other complications or that the selected treatment will result in a certain outcome make clinical decision-making a challenging task. Bayesian networks have been proven to be a strong tool to model the complex dependence between many factors [8] and capture the way an expert understands the relationships among all the features [9]. Bayesian network is characterized by several capabilities: (1) model complex problems with causal dependencies where a significant degree of uncertainty is involved, (2) combine multiple sources of information including data and experts’ judgment, (3) present as an interpretable graphical structure, and (4) model interventions and reason both diagnostically and prognostically [10]. Bayesian network modeling is widely used in fields like clinical decision support [11], interactions between multiple diseases [12], and also in diagnostic diseases [13,14]. Previous research suggested that predictions from Bayesian network models perform better than competing approaches such as stepwise logistic regression [15]. Tevis et al [16] applied the Bayesian network to visualize the relationships among multiple perioperative complications to identify those complications most strongly associated with adjacent complications. Shen et al [7] also used the Bayesian network model to construct and quantify the association network among multiple perioperative complications and to elucidate the possible evolution trajectories. Cardiac surgery is characterized by the high risk of cardiovascular and other complications [17]. Reducing perioperative complications is key to improving the prognosis of patients undergoing heart valve surgery. However, few studies have revealed and quantified the possible progression trajectories of perioperative complications in patients with VHD.

Hence, the focus of our study was to construct and quantify the association network among multiple perioperative complications to investigate whether we could identify predictable patterns of secondary complication occurrences in the postoperative period after the first complication based on the Bayesian network model. We aimed to explore critical pathways in the network to support the development of targeted postoperative prevention strategies in patients with VHD.

## Methods

### Data Sources and Patients

The patients who underwent valvular surgery were identified from the Chinese Cardiac Surgery Registry (CCSR) database from January 2016 to December 2018. The CCSR database prospectively collected in-hospital and 30-day outcomes data on valvular surgery patients from 94 hospitals nationwide [18]. Patients were included in the study if they underwent valvular surgery procedures or valve surgery combined with coronary artery bypass graft surgery (CABG). Patients with missing information on perioperative complications and those who have not undergone cardiopulmonary bypass (CPB) were excluded from this study.

### Perioperative Complications

We selected 12 perioperative complications, prolonged intensive care unit (ICU) stays (defined as exceeding the 75th percentile for ICU length of stay [19]), prolonged mechanical ventilation (defined as more than 24 h by the Society of Thoracic Surgeons [STS] guidelines [20]), increased chest tube drainage (defined as exceeding the 75th percentile for chest tube drainage), postoperative AF, postoperative myocardial infarction (postoperative myocardial infarction [MI], defined as new-onset pathologic Q-wave or left bundle branch block on the 12-lead electrocardiogram [21]), sternal wound infections, pericardial tamponade, reoperation, secondary tracheal intubation, stroke (defined as an acute episode of focal or global neurologic dysfunction caused by brain, spinal cord, or retinal vascular injury as a result of hemorrhage or infarction in which the neurologic dysfunction lasts for more than 24 h), postoperative renal failure (RF), multiple organ dysfunction syndrome (MODS). Among the 12 outcomes, 6 outcomes were found in the Society of Thoracic Surgeons database, including prolonged mechanical ventilation, postoperative AF, sternal wound infections, reoperation, stroke, postoperative RF [22]. In addition, prolonged ICU stays, increased chest tube drainage, postoperative MI, and pericardial tamponade have been demonstrated to be associated with increased perioperative morbidity and mortality after cardiac surgery [23-26]. Secondary tracheal intubation is a common but critical issue in the management of patients requiring mechanical ventilation in the ICU [27], which may further worsen the overall prognosis of patients. MODS is also the major cause of mortality after heart valve surgery [28]. Therefore, the types of complications we included are representative and reasonable. Death was defined as death occurring during the hospitalization and after discharge from the hospital but before the end of the 30th postoperative day.

### Statistical Analysis

We calculated the proportion of common complications of valvular surgery. The number of deaths and the mortality rate for patients with each complication were also calculated to reflect the severity of different complications. The rate of co-occurring complications was defined as the percentage of patients that developed each complication and who also had other complications. The phi coefficient (phi) quantified the degree of concordance between any two complications. We used the rate of co-occurring complications and the phi to identify the co-occurrence relationships between complications. In addition, we compared the perioperative characteristics between patients with and without perioperative complications using the *χ*^*2*^ test. R software (version 4.2.3; R Foundation for Statistical Computing) was used for statistical analyses.

### Construction and Evaluation of a Complications Network

To investigate further interactions among perioperative complications, we applied a Bayesian network to patients with at least one complication. A Bayesian network is a graphical representation of a joint probability distribution. Learning a Bayesian network implies two tasks: (1) structure learning: identifying the topology of the Bayesian network and (2) parametric learning: estimating numerical parameters (conditional probabilities) given a network topology [29].

During the structure learning stage, we constructed a directed acyclic graph (DAG) to describe the dependencies between these nodes. The nodes from which an arrow pointed were referred to as the “parent” nodes and the nodes pointed to by an arrow were referred to as “child” nodes. The state of the “parent” nodes affects their “child” nodes [30]. Taking into account the forbidden directions of the arcs (from death to other complications), we adopted the hill-climbing algorithm based on a score search to create a Bayesian network [31]. In addition, we used bootstrap resampling (1000 replicates, each with a resultant candidate network structure) to obtain a stable network structure. The arcs with strength probability >0.75 and direction probabilities ≥0.5 were considered finally.

Parameter values were obtained by using the maximum likelihood algorithm in the parameter learning phase [32]. The parameters are used to describe the probability distribution for each node, based on which we calculated the probability of a complication predicted using the Bayesian network. When one or more complications occurred, the probability of the occurrence of any complication was predicted, which we used to quantify the association between complications.

All analyses were performed using R software, including the *UpSet*, *bnlearn,* and *gRain* packages.

### Ethical Considerations

The CCSR database was generated in 2013, including 87 large cardiac centers across the country with routine data submission, strict data audits, and instant feedback. These sites are the leading cardiac centers in local regions and have many features that are common among large cardiac care centers in China. The study was conducted in accordance with the Declaration of Helsinki and approved by the Institutional Review Board of Fuwai Hospital (protocol code 2021‐1477; date of approval is August 11, 2021). Informed consent was obtained from all subjects involved in the study. Data were collected and analyzed using a pseudonymous form. The participants did not receive any form of financial compensation for participating in this trial.

## Results

### Characteristics of the Participants

Based on inclusion and exclusion criteria, 37,285 patients were included in the analysis. Table 1 shows that we compared the perioperative characteristics of patients with at least one complication with those without. Nearly all assessed perioperative characteristics showed significant differences between groups (*P*<.05). Compared with patients who did not develop perioperative complications, patients with at least one complication were more likely to be male and aged 65 years and older (2724/18,700, 14.6% vs 4686/18,585, 25.2%), have more comorbidities, and have longer CPB time (7539/18,700, 40.3% vs 11,102/18,585, 59.7%) and aortic cross-clamp (ACC) time (7701/18,700, 41.2% vs 10,702/18,585, 57.6%).

**Table 1.** Baseline characteristics of the participants in each sample.

Characteristic	Total, n (%)	Without complication (N=18,700), n (%)	With complication (N=18,585), n (%)	*P* value
Demographics				
Age ≥65 years	7410 (19.9)	2724 (14.6)	4686 (25.2)	<.01
Female	17,640 (47.3)	9353 (50)	8287 (44.6)	<.01
BMI ≥30	1147 (3.1)	538 (2.9)	609 (3.3)	.03
Comorbidity				
Smoking	14,901 (40.0)	7766 (52.1)	7135 (38.4)	<.01
Diabetes mellitus	2494 (6.7)	972 (5.2)	1522 (8.2)	<.01
Hypertension	9875 (26.5)	4402 (23.5)	5473 (29.4)	<.01
CRF^a^	1140 (3.1)	605 (3.2)	535 (2.9)	.05
COPD^b^	342 (0.9)	114 (0.6)	228 (1.2)	<.01
Peripheral vascular disease	530 (1.4)	240 (1.3)	290 (1.6)	.03
Stroke	1605 (4.3)	705 (3.8)	900 (4.8)	<.01
Heart failure	2286 (6.1)	901 (4.8)	1385 (7.5)	<.01
Arrhythmia	8883 (23.8)	3898 (20.8)	4985 (26.8)	<.01
Myocardial infarction	758 (2)	173 (0.9)	585 (3.1)	<.01
Symptomatic coronary artery disease	2722 (7.3)	812 (4.3)	1910 (10.3)	<.01
Echocardiography results				
EF^c^<50	3870 (10.4)	1464 (7.8)	2406 (12.9)	<.01
LVEDD^d^^,^^e^	18,768 (50.3)	9181 (49.1)	9587 (51.6)	<.01
LAD^f^^,^^g^	28,319 (76)	13,884 (74.2)	14,435 (77.7)	<.01
TRV^h^>2.9 m/s	12,540 (33.6)	5893 (31.5)	6647 (35.8)	<.01
Biochemical indexes				
High creatinine^i^	5935 (15.9)	2474 (13.2)	3461 (18.6)	<.01
TC^j^>5.2 mmol/L	5670 (15.2)	2877 (15.4)	2793 (15)	.34
LDL^k^>3.4 mmol/L	5501 (14.8)	2894 (15.5)	2607 (14)	<.01
CPB^l^				
Prolonged CPB time^m^	18,641 (50)	7539 (40.3)	11,102 (59.7)	<.01
Prolonged ACC^n^ time^o^	18,403 (49.4)	7701 (41.2)	10,702 (57.6)	<.01
Mortality	816 (2.2)	82 (0.4)	734 (3.9)	<.01

a CRF: chronic renal failure.

b COPD: chronic obstructive pulmonary disease.

c EF: ejection fraction.

d LVEDD: left ventricular end-diastolic diameter.

e LVEDD: for women, LVEDD >50; for men, >54.

f LAD: left atrial diameter.

g LAD: for women, LAD >37; for men, >39.

h TRV: tricuspid regurgitation velocity.

i High creatinine: for women, creatinine >84 mmol/L; for men, >104 mmol/L

j TC: total cholesterol.

k LDL: low-density lipoprotein.

l CPB: cardiopulmonary bypass time.

m Prolonged CPB time: Prolonged CPB time was defined as CPB time longer than the median of the overall cohort.

n ACC:

o Prolonged ACC time: Prolonged ACC time was defined as ACC time longer than the median of the overall cohort.

### Complication Occurrence and Mortality

In total, 18,585 patients developed at least one complication. Table 2 shows the frequency and mortality for each complication. Prolonged ICU stays, prolonged mechanical ventilation, and increased chest tube drainage were the 3 most common complications, occurring in nearly half of patients, while patients with these complications had the lowest mortality rate, accounting for 4.8% (n=448), 4.9% (n=460), and 6.8% (n=579), respectively. Our results showed that sternal wound infections and stroke were least common in valvular disease patients undergoing cardiopulmonary bypass (0.5%), but were associated with higher mortality (20.2% [n=19] and 35.9% [n=33], respectively). The mortality rate for patients with different complications varied widely. Except for MODS, postoperative RF was the more common complication with the highest mortality rate (n=223, 49.2%).

**Table 2.** Frequency and mortality of individual complications.

Complications	Total patients with complication, n (%)	Deaths after complication, n (%)
Increased chest tube drainage	9312 (50.1)	448 (4.8)
Prolonged ICU^a^ stays	9321 (50.2)	460 (4.9)
Prolonged mechanical ventilation	8489 (45.7)	579 (6.8)
Postoperative AF^b^	484 (2.6)	39 (8.1)
Postoperative MI^c^	181 (1)	15 (8.3)
Sternal wound infections	94 (0.5)	19 (20.2)
Pericardial tamponade	163 (0.9)	33 (20.2)
Reoperation	1068 (5.7)	174 (16.3)
Secondary tracheal intubation	537 (2.9)	202 (37.6)
Stroke	92 (0.5)	33 (35.9)
Postoperative RF^d^	453 (2.4)	223 (49.2)
MODS^e^	299 (1.6)	274 (91.6)

a ICU: intensive care unit.

b AF: atrial fibrillation.

c MI: myocardial infarction.

d RF: renal failure.

e MODS: multiple organ dysfunction syndrome.

### Co-Occurrence of Complications

**Figure 1.**
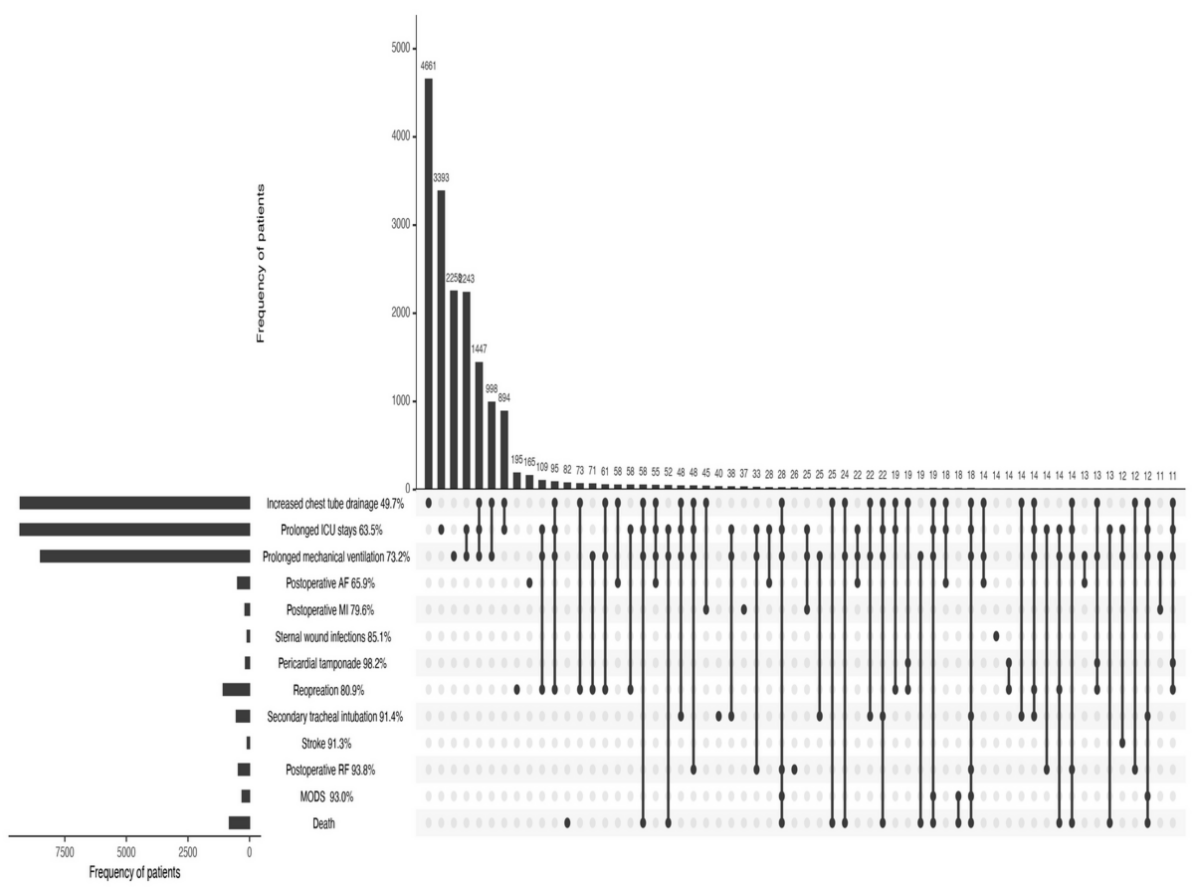
UpSet plot of the co-occurrence of complications. The number of each complication is represented on the left bar plot. Possible co-occurrence between complications is represented by dark circles, and their number is shown on the top bar plot. The rate of co-occurrence is shown on the left side of the dark circles. ICU: intensive care unit; AF: atrial fibrillation; MI: myocardial infarction; RF: renal failure; MODS: multiple organ dysfunction syndrome.

**Figure 2.**
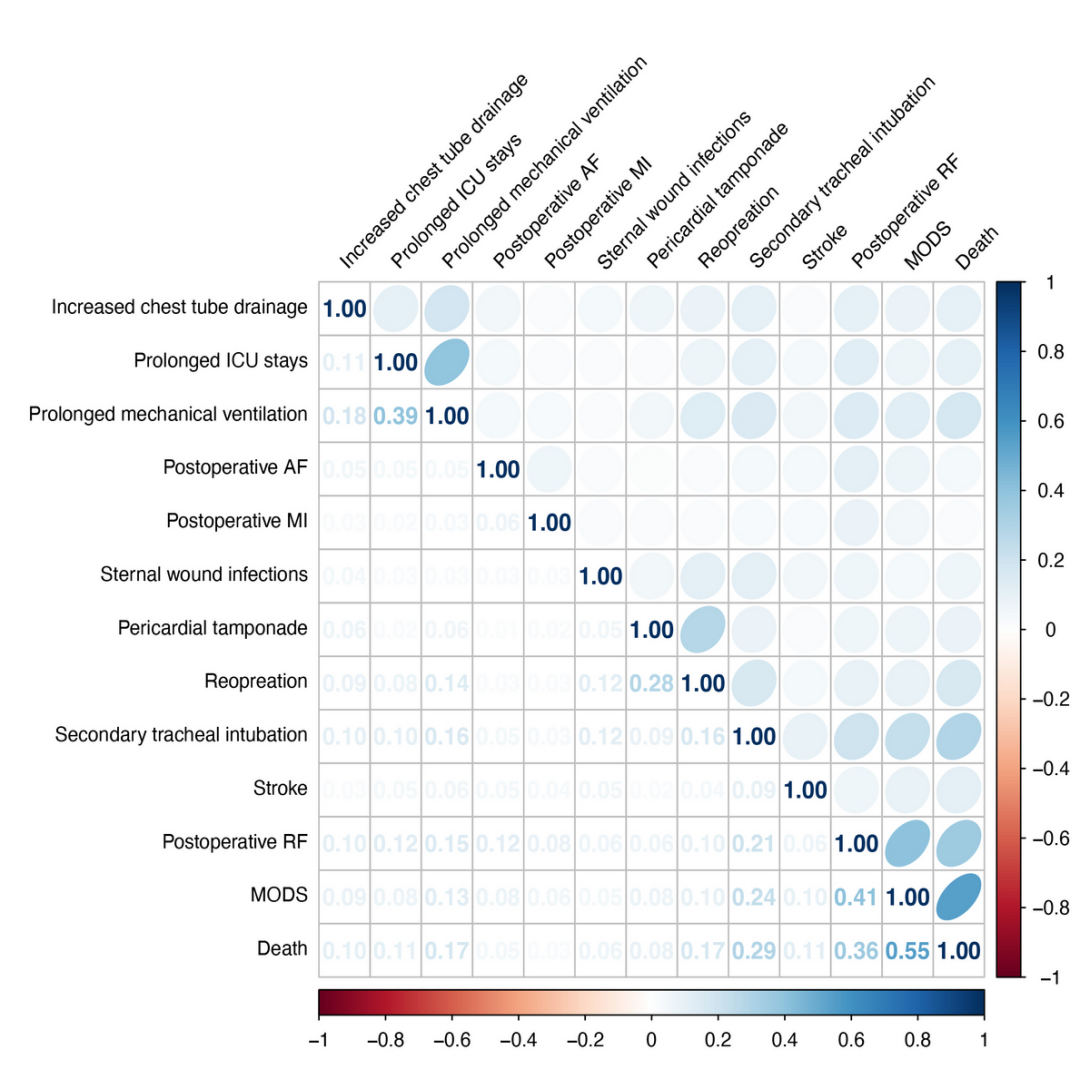
The heat map of the phi coefficients between complications. ICU: intensive care unit; AF: atrial fibrillation; MI: myocardial infarction; RF: renal failure; MODS: multiple organ dysfunction syndrome.

The UpSet plot in Figure 1 describes the co-occurrence of 12 complications in the dataset, with the rate of co-occurrence on the left side. More than 40% of the participants who experienced complications had 2 or more complications. Nearly 90% of patients who died had 2 or more complications. The rate of co-occurrence of nearly all complications was more than 60%. Especially for some serious complications, such as stroke, postoperative RF, sternal wound infections, and MODS, tended to occur in clusters. The rate of co-occurrence of these complications exceeded 90%. Similarly, the correlation analysis showed the strong associations between postoperative RF, sternal wound infections, and MODS (see Figure 2). The strongest correlation was between postoperative RF and MODS (phi=0.41). Although some complications with lower mortality were milder, such as prolonged ICU stays, prolonged mechanical ventilation, and increased chest tube drainage, the rate of co-occurrence of these complications was also higher (63.5%, 73.2%, and 49.7%, respectively). These complications appeared to be the underlying factors for subsequent complications.

### Construction of the Bayesian Network of Perioperative Complications

**Figure 3.**
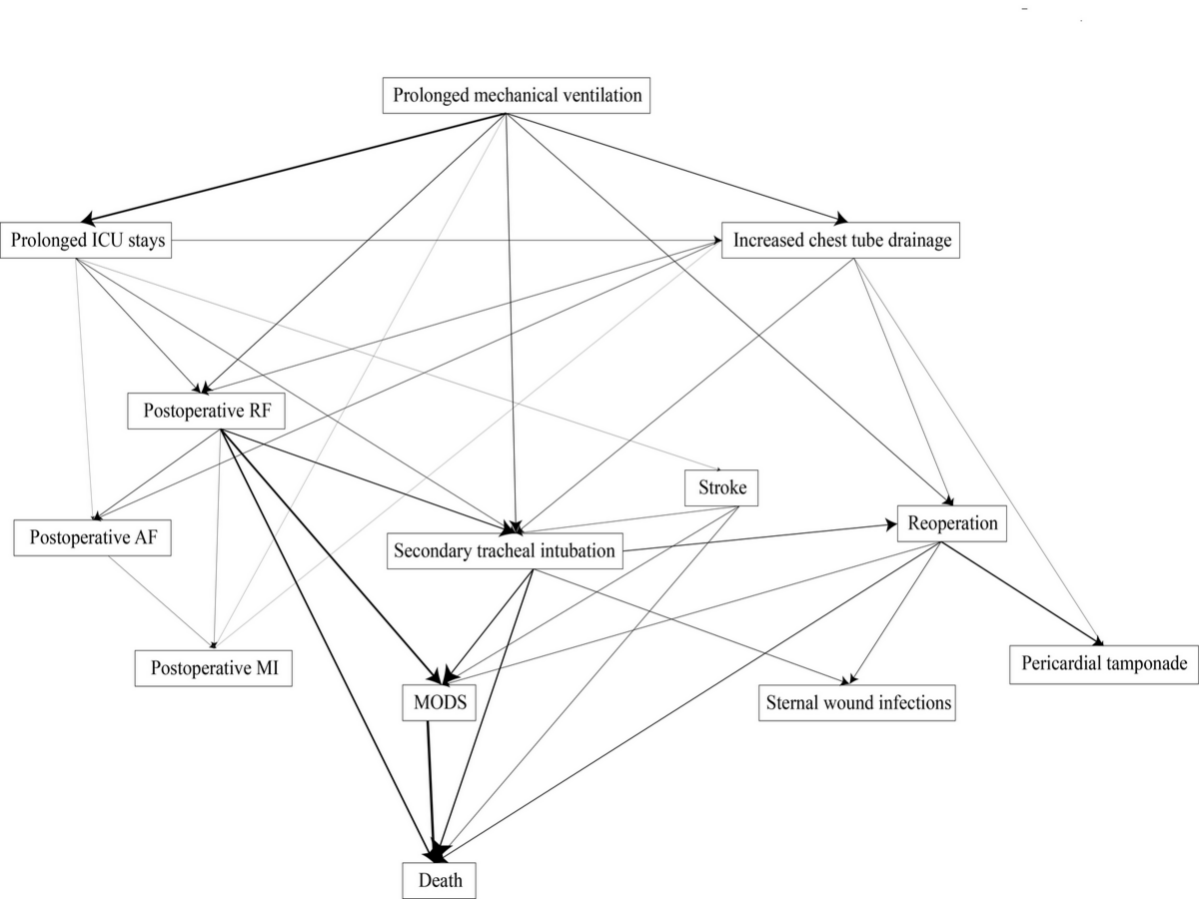
Bayesian network of the complications and death. As the arc becomes thicker, the phi coefficient between the connected complications increases. ICU: intensive care unit; AF: atrial fibrillation; MI: myocardial infarction; RF: renal failure; MODS: multiple organ dysfunction syndrome.

Figure 3 shows the Bayesian network modeling the relationships among complications in patients who developed complications. Each node in the figure represents a complication, and the arrows represent direct relationships among complications. There were 13 nodes and 34 directed arcs in the Bayesian network. All the arcs had a strength >0.85 and 30/34 arcs (88.2%) had a direction probability >0.70 (Multimedia Appendix 1). We identified complex relationships between multiple complications. In addition, by calculating the Markov blanket, we identified the critical complications of the network, namely secondary tracheal intubation, stroke, reoperation, and postoperative RF. Prolonged ICU stays, prolonged mechanical ventilation, and increased chest tube drainage appeared to be the starting points for these critical complications. By affecting the occurrence of postoperative RF, stroke, secondary tracheal intubation, and reoperation, prolonged ICU stays, prolonged mechanical ventilation, and increased chest tube drainage led indirectly to death, forming the path from primary complications to critical complications, ultimately leading to death. Postoperative AF, postoperative MI, sternal wound infections, and pericardial tamponade had no direct arc connected with death. In addition, there were more positively correlated arcs between serious complications than other complications, such as the strong relationships between the complications that could be connected to death and MODS, reflecting the cascading effect between complications.

### Probability Prediction Using Complications Network

The parameters (probability) of the Bayesian network were learned from the dataset containing 13 variables and 37,285 patients. When providing additional information regarding patients’ complications, the probability of each complication occurring in the network can be automatically updated. Therefore, we calculated the probability of the occurrence of other complications and death, given the occurrence of 1 complication (see Table 3). The probability of death of more than 40% was the complications consisting of secondary tracheal intubation, stroke, postoperative RF, and MODS. Except for stroke, there is a close relationship between secondary tracheal intubation, postoperative RF, and MODS. When any one of these complications occurred, the calculated probability of the other complication is around 20% or more. In addition, we found that postoperative MI, sternal wound infections, and stroke were less likely to occur secondary to other complications. Given the occurrence of other complications, the probability of postoperative MI, sternal wound infections, and stroke was all below 10%. The occurrence of these complications appeared to be mainly associated with the preoperative or intraoperative factors. We also calculated the probability of death when assuming that more than 1 complication co-occurred (see Table 4). When one complication eventually caused MODS, the mortality rate exceeded 90%.

**Table 3.** The probability of the occurrence of the complications given the occurrence of one complication (%).

Interested complications	Evidence provided to the Bayesian network, %											
	Increased chest tube drainage	Prolonged ICU^a^ stays	Prolonged mechanical ventilation	Postoperative AF^b^	Postoperative MI^c^	Sternal wound infections	Pericardial tamponade	Re-operation	Secondary tracheal intubation	Stroke	Postoperative RF^d^	MODS^e^
Increased chest tube drainage	—^f^	31.6	38.9	51.6	48	51.5	68.6	49.1	63	40	64.6	52.2
Prolonged ICU stays	32.1	—	55.4	48.4	38	51.5	45.1	48.4	59.1	66.7	66.1	55.6
Prolonged mechanical ventilation	36.3	51	—	40.6	50	60.6	51	62.6	76.2	52.4	74.8	62.2
Postoperative AF	3.3	3	2.8	—	16	4.3	5.9	5.1	4.4	0	14.3	7.3
Postoperative MI	1	0.8	1.1	5.2	—	0	2	1.5	1.1	0	7.1	2.3
Sternal wound infection	0.7	0.7	0.9	0.8	0	—	2	5.5	7.7	0	2.4	2.1
Pericardial tamponade	1.4	0.9	1.1	2.3	2.3	5.3	—	13.6	4.3	3.3	2.6	3.4
Reoperation	5.5	5.3	7.5	9	8	45.5	72.5	—	23.2	9.5	22.8	16.7
Secondary tracheal intubation	4.7	4.3	6.1	6.6	4.8	42.4	11.8	15.4	—	3.3	27.6	30
Stroke	0.5	0.6	0.5	0	0	0	2	0.7	0.7	—	0.7	5.6
Postoperative RF	3.4	3.4	4.2	12.9	18	9.1	7.8	10.6	19.3	4.8	—	63.3
MODS	1.9	2	2.5	0.8	3.3	10	6.5	5.5	19.1	16.7	37.7	—
Death	4.7	4.9	5.7	8.1	7	15	15.2	21.2	46.9	47.6	57	96

a ICU: intensive care unit.

b AF: atrial fibrillation.

c MI: myocardial infarction.

d RF: renal failure.

e MODS: multiple organ dysfunction syndrome.

f Not available.

**Table 4.** The probability of death given the occurrence of multiple complications.

Evidence provided to the Bayesian network			
Complication 1 (The probability of death when Complication 1 occurs, %)	Complication 2	Complication 3	The probability of death when complications 1‐3 occur (%)
Prolonged ICU stays (4.9)	Stroke		42.9
	Stroke	Secondary tracheal intubation	58.6
	Stroke	MODS	99.5
	Secondary tracheal intubation		48.3
	Secondary tracheal intubation	Reoperation	57.1
	Secondary tracheal intubation	MODS	90.1
	Postoperative RF		57.0
	Postoperative RF	Secondary tracheal intubation	79.2
	Postoperative RF	MODS	93.3
Increased chest tube drainage (4.7)	Reoperation		26.4
	Secondary tracheal intubation		50.0
	Secondary tracheal intubation	Reoperation	53.3
	Secondary tracheal intubation	MODS	92.3
	Postoperative RF		61.9
	Postoperative RF	Secondary tracheal intubation	79.2
	Postoperative RF	MODS	97.7
Prolonged mechanical ventilation (5.7)	Reoperation		25.1
	Secondary tracheal intubation		46.7
	Secondary tracheal intubation	Reoperation	50.0
	Secondary tracheal intubation	MODS	93.3
	Postoperative RF		57.5
	Postoperative RF	Secondary tracheal intubation	78.1
	Postoperative RF	MODS	94.2

a ICU: intensive care unit.

b Not available.

c MODS: multiple organ dysfunction syndrome.

d RF: renal failure.

## Discussion

### Principal Findings

In this study, we constructed, for the first time, a new Bayesian network-based model of 12 perioperative complications and death from 37,285 VHD patients undergoing valve surgery in the CCSR. According to the network model, we demonstrated possible associations and visualized development paths between perioperative complications and quantified the pathways of complication occurrence and progression. Therefore, we identified critical pathways for the development of complications associated with valve surgery, which help to explore ways to halt the cascade of complications in patients undergoing the valve surgery in our future work. Different from previous studies, our finding provides an intuitive perspective that emphasizes the overall correlation for improving the prevention strategies of perioperative complications of VHD.

The complication network describes the dependencies between perioperative complications after valve surgery. The complication in the network may represent a point of intervention. The associations among complications provide the basis for the measures that will prevent or interrupt the occurrence of more serious complications. Our network analysis showed that increased chest tube drainage might contribute to the development of postoperative AF and postoperative RF, and prolonged mechanical ventilation increases the risk of acute kidney injury. The work is consistent with evidence for the role of higher drainage volume on the emergence of postoperative acute kidney injury and postoperative AF [33,34]. Kimura et al [35] also demonstrated that in pediatric cardiac patients, cardiac surgery-associated acute kidney injury was associated with prolonged mechanical ventilation. While discovering the logical relationship between complications, we found stroke appears to be less related to other serious complications. Although previous studies have concluded that patients with postoperative AF have an increased risk of stroke, Rasmussen et al [36] demonstrated that there was no difference in the adjusted risk of postoperative stroke in postoperative AF versus non‐postoperative AF patients, which agreed with our results. The distinct trajectory for stroke suggests that postoperative factors may not be directly associated with the occurrence of stroke. For the prevention of stroke, it may be more appropriate to intervene in preoperative or intraoperative factors. In addition, we found that the association between serious complications was stronger. When secondary tracheal intubation, postoperative RF, and MODS occurred, the probability of any other of these complications occurring was higher. Therefore, when rescuing a certain organ, joint prevention and intervention measures must be formulated to protect the functions of other organs and minimize the compounding effects of multiple complications [37].

We also explored the synergistic effect of multiple complications on mortality. An increase in the number of multiple complications shows a positive cumulative effect, suggesting the importance of special measures for serious complications such as real-time monitoring. In addition, we calculated mortality in the presence of multiple complications. Therefore, the network could be used to inform real-time risk prediction models that alert surgical teams to the changing risks of associated events for individual patients. For example, patients with postoperative RF or stroke may require careful selection of secondary tracheal intubation to avoid progression to more serious consequences. Our analysis suggests that for patients with valvular disease undergoing surgery, the prediction and prevention of complications may meaningfully extend into the postoperative period. Strategies based on the postoperative events could significantly improve our predictive capabilities and thus initiate strategies to reduce secondary complications.

In the past, it has been witnessed that some studies have applied network models to explore the complex relationships underlying a wide range of perioperative complications [16,38]. These studies initially found the relationship between perioperative complications. However, complication networks in the above studies are defined pairwise associations between diseases, which might miss important synergistic relationships in data. The uncertainties, including the progress of complications in individual patients, hospital work-flow management, and other factors, render an overall understanding hard to achieve. Now, our attempt provides a starting point to implement network theory to dissect the mechanistic basis of complex perioperative complications of cardiac surgery, from which to generate new discoveries traditional approaches fail to identify.

Our study had several limitations. First, the complications we included are not comprehensive. In the future, we will include more perioperative complications, such as paravalvular leakage and blood transfusion, to reveal the development process of perioperative complications. In addition, we will also include the information of postdischarge complications to improve completeness of research of complications. Second, our data lacks the ability to sequence complication, which prevents further confirmation of the causality. In the follow-up, we will collect the cohort recording the time of complication occurrence and further explore the associations of perioperative complication development using dynamic Bayesian networks. Third, high collinearity between variables will affect Bayesian network performance, including inaccurate parameter estimation, increased model complexity, unreliable inference results, and difficulty in structure learning. We evaluated the multicollinearity between variables, and the results showed that the Pearson correlation coefficients between all variables were below 0.6 (Multimedia Appendix 1). The highest Pearson correlation coefficient was 0.55, between MODS and mortality. In addition, we performed eigenvalue analysis and calculated the condition number (5.660998, with a condition number greater than 30 indicating severe multicollinearity). The results indicate that the variables are linearly independent, and multicollinearity is minimal. In any case, the role of the network model is predicated on a tremendous advance in profiling the complex complication relationship. The model may also find more promising applications in analyzing the comprehensive impact of preoperative impact on multiple complications and the design of more personalized prevention of perioperative complications.

### Conclusions

In conclusion, we have created a Bayesian network that elucidates the underlying relationships and evolving progress of complications in valve surgery, and we have quantified the associations between 12 complications, which provides a basis for the development of targeted preventive measures to prevent further deterioration in patients undergoing valve surgery.

## Supplementary material

10.2196/68710 Multimedia Appendix 1 Supplementary tables.

## Abbreviations

AF: atrial fibrillation
CCSR: China Cardiac Surgery Registry
CPB: cardiopulmonary bypass
ICU: intensive care unit
MI: myocardial infarction
MODS: multiple organ dysfunction syndrome
RF: renal failure
VHD: valvular heart disease
