# Supplementary material for: Probing the Relationship Between Perioperative Complications in Patients With Valvular Heart Disease: Network Analysis Based on Bayesian Network
Source: JMIR Form Res. 2025 Oct 7;9:e68710. doi: 10.2196/68710 (PMC12503447; doi:10.2196/68710)
Supplement: Multimedia Appendix 1 [file formative-v9-e68710-s001.docx]

**Table 4**

| Evidence provided to the Bayesian network | |  |  |  |  | |  |  |
| --- | --- | --- | --- | --- | --- | --- | --- | --- |
| Complication 1  (The probability of death when Complication 1 occurs, %) | Complication 2 | | Complication 3 | | | The probability of  death when complication 1-3 occur (%) | | |
|  |  |  |  |  |  |  |  |  |
| Prolonged ICU  stays (4.9) | Stroke |  |  |  | | 42.9 |  |  |
|  | Stroke |  | Secondary tracheal intubation | | | 58.6 |  |  |
|  | Stroke |  | MODS |  | | 99.5 |  |  |
|  | Secondary tracheal intubation | |  |  | | 48.3 |  |  |
|  | Secondary tracheal intubation | | Reoperation | | | 57.1 |  |  |
|  | Secondary tracheal intubation | | MODS |  | | 90.1 |  |  |
|  | Postoperative RF | |  |  | | 57.0 |  |  |
|  | Postoperative RF | | Secondary tracheal intubation | | | 79.2 |  |  |
|  | Postoperative RF | | MODS |  | | 93.3 |  |  |
| Increased chest  tube drainage (4.7) | Reoperation |  |  |  | | 26.4 |  |  |
|  | Secondary tracheal intubation | |  |  | | 50.0 |  |  |
|  | Secondary tracheal intubation | | Reoperation | | | 53.3 |  |  |
|  | Secondary tracheal intubation | | MODS |  | | 92.3 |  |  |
|  | Postoperative RF | |  |  | | 61.9 |  |  |
|  | Postoperative RF | | Secondary tracheal intubation | | | 79.2 |  |  |
|  | Postoperative RF | | MODS |  | | 97.7 |  |  |
| Prolonged  mechanical ventilation (5.7) | Reoperation |  |  |  | | 25.1 |  |  |
|  | Secondary tracheal intubation | |  |  | | 46.7 |  |  |
|  | Secondary tracheal intubation | | Reoperation | | | 50.0 |  |  |
|  | Secondary tracheal intubation | | MODS |  | | 93.3 |  |  |
|  | Postoperative RF | |  |  | | 57.5 |  |  |
|  | Postoperative RF | | Secondary tracheal intubation | | | 78.1 |  |  |
|  | Postoperative RF | | MODS |  | | 94.2 |  |  |
